# Clinical findings associated with diagnosing lumbar foraminal stenosis: systematic review

**DOI:** 10.1186/s12891-026-10614-5

**Published:** 2026-11-03

**Authors:** Harald Ekedahl, Malin Eleonora av Kák Gustafsson, Caroline Larsson

**Affiliations:** Department of health sciences, Faculty of Medicine, Lund University, Lund, Sweden

**Keywords:** Back pain, Diagnostic imaging, Lumbar radiculopathy, Spine

## Abstract

**Purpose:**

To determine the diagnostic value of history taking and clinical examination tests for diagnosing lumbar radicular pain due to foraminal stenosis (FS) as established using magnetic resonance imaging (MRI).

**Method:**

An electronic search of PubMed, CINAHL, Embase and MEDLINE was conducted. Studies with diagnostic accuracy of FS using dichotomous index tests (patient history measures and clinical examination tests) were included. The risk of bias was assessed using the Quality Assessment of Diagnostic Accuracy Studies-2 (QUADAS 2) tool. Diagnostic value of index test was analyzed using proportions of positive findings (sensitivity). Specificity was not obtained. The results of all patients with FS were compared with all patients of a different specific diagnosis, disc herniation (DH) or central spinal stenosis (CSS), by comparing the proportion of positive findings for each index test.

**Results:**

A total of 2,491records were identified. After title and abstract screening, 1,951 records were excluded, and 84 full-text articles were assessed for eligibility. Finally, 15 studies were included. No study investigating accuracy of tests for patients with recess stenosis was found. In the included studies, 737 subjects had FS, and 10 different index tests were found (2 patient history measures and 8 clinical examination tests). Of the 15 included studies, overall risk of bias was judged as low in two studies, while remaining studies had some concerns primarily due to unclear risk in the index test and flow and timing domains. Applicability concerns were generally low across studies. The index tests with highest average proportion of positive findings in detecting FS were Leg pain at rest (74%; 4 studies, *n* = 190) and Lumbar extensions test (66%; 11 studies, *n* = 537); and the test with the lowest proportion of positive findings was Straight leg raise (SLR; 33%; 10 studies, *n* = 471).

**Conclusion:**

This review concluded that SLR has the lowest ability to detect lumbar foraminal stenosis, whereas positive Lumbar extension test and Leg pain at rest have the highest ability to detect the diagnosis. These findings should be interpreted cautiously given methodological uncertainties and limited diagnostic accuracy evidence.

**Supplementary Information:**

The online version contains supplementary material available at https://doi.org/10.1186/s12891-026-10614-5.

## BACKGROUND

To diagnose lumbar radicular pain, clinicians evaluate the likelihood of radiculopathy (compression of the nerve root) through history taking and clinical examination and then magnetic resonance imaging (MRI) to verify the hypothesis. The cause of unilateral radicular symptoms and signs can be malignant, but more often due to degenerative changes such as disc herniation or foraminal stenosis [1]. These degenerative changes can occur without radicular symptoms and are frequently seen on MRI [1]. Thus, guidelines suggest clinical examination along with MRI as the most relevant assessment when the radiating leg pain is persistent and causes major disability [2].

The clinical assessment of subjects with lumbar disc herniation has been described in detail [3] and has adequate accuracy [4], however, the clinical examination to diagnose lumbar foraminal or recess stenosis is not well understood and poorly investigated. This means there is a risk that patients with lumbar foraminal stenosis (FS) are incorrectly assessed in primary care, the diagnosing procedure is inaccurate, and the surgical decision-making may be based on uncertain clinical findings. Systematic reviews with meta-analysis have been conducted regarding the accuracy of history taking and clinical examination tests for the diagnosis of disc herniation (DH) and central spinal stenosis (CSS) [4, 5]. Studies of clinical examination tests have stated that the diagnostic accuracy of the tests differs between different radicular pain diagnoses [6, 7]; however, no systematic review with meta-analysis has been conducted for FS.

## Objectives

To determine the diagnostic value of history taking and clinical examination tests for diagnosing lumbar radicular pain due to foraminal stenosis (FS) as established using magnetic resonance imaging (MRI).

## Methods

### Protocol registration

The study procedure follows the PRISMA-DTA (Preferred Reporting Items for Systematic Reviews and Meta-Analysis Protocols for diagnostic test accuracy studies) reporting guidelines [8] (Appendix 1). The study protocol was pre-registered in PROSPERO (CRD42024559813).

### Eligibility criteria

#### Type of studies and participants

Published studies that compare clinical tests and patient history with the stated reference standard (MRI) were included. Any quantitative study design using diagnostic accuracy data or correlating data or examining associations between dichotomous variables was eligible.

Patient Inclusion: Adults (≥ 18 years) with low back pain (LBP) and radiating unilateral symptoms or radiculopathy; acute, sub-acute, or chronic symptoms; clinically assessed in either primary care or hospital care units.

Studies were excluded if they were not written in English; did not compare an index test (patient history and/or clinical examination) with a reference standard for identifying foraminal (FS); or evaluated screening tools in which symptoms and tests were combined to a composite score (e.g. The Leeds assessment of neuropathic symptoms and signs or Oswestry disability index).

#### Index Tests

Studies evaluating patient history and clinical examination tests were eligible. Patient history was defined as any recorded or self-reported symptoms associated with unilateral radicular pain such as walking distance due to leg symptoms, standing time in minutes and pain location. Clinical examination tests were defined as any physical assessments used to support the diagnosis of FS, including neurodynamic tests, neurologic examination, range of motion assessment and pain provocation test. Only dichotomous measures were included.

#### Reference standards

MRI was used as the primary reference standard. Studies using MRI and additional imaging modalities were also accepted. More than one MRI system exists for the diagnosis of lumbar FS. It is generally agreed upon that foraminal nerve compression is assessed using sagittal images described by Lee et al. In this system, distortion of the nerve root, and grade III when distortion or other morphologic change in the nerve root is evident. Grades II and III are considered as high-grade nerve compression and grade I applies when perineural fat is obliterated in 2 opposing directions (vertical or transverse), grade II when perineural fat is obliterated in 4 directions without morphologic verify the diagnosis of FS [9]. However, other similar MRI assessment systems were accepted. In cases where the compression was caused by a combination of degenerative changes and disc herniation (DH), the cases were disregarded as FS.

### Information source

PubMed, Embase, CINAHL, and MEDLINE were searched from start of database until October 2024. In addition to the electronic database search, other potentially eligible studies were identified by reviewing the reference lists of each of the eligible articles. An additional search with identical search terms was conducted from October 2024 to September 2026.

### Search

The search strategy combined standardized keywords, such as MeSH and Emtree terms, and specific terms related to lumbar FS and clinical diagnostic tests. Boolean operators (AND/OR) were used to combine search terms. The search strategy was adapted for each database. The full search strategy is provided in Appendix 2.

### Study selection

Study selection was conducted in two stages using the Covidence systematic review software, (Veritas Health Innovation, Melbourne, Australia). First, two reviewers (HE and CL) independently screened titles and abstracts against the study aim and predefined eligibility criteria. Consensus was reached before proceeding to full-text review. Second, the full texts of potentially eligible articles were assessed independently by the same reviewers using the same criteria, and consensus was again achieved. When consensus could not be reached at any stage, a third reviewer (MG) was consulted. Reasons for full-text exclusions were stated.

### Data collection process

Two independent reviewers (HE and CL) independently extracted data from each included study using a standardized data extraction form. The following data were extracted: study design and setting, baseline demographic characteristics, number of participants, target condition (FS), comparative groups (e.g., DH or central spinal stenosis (CSS)), reference standards and index tests (patient history and clinical examination). When available, frequencies of positive and negative findings were collected to enable calculation of diagnostic value estimates. Extracted data were cross-checked and discrepancies were resolved by consensus. Disagreements were resolved through discussion or consultation with a third reviewer (MG).

### Risk of bias and applicability

Risk of bias and applicability concerns were independently assessed by two reviewers using the Quality Assessment of Diagnostic Accuracy Studies-2 (QUADAS-2) tool [10]. The tool consists of four key domains: patient selection, index test, reference standard, and flow/timing. Each domain was judged as having low, high, or unclear risk of bias [10]. An overall risk-of-bias judgment was additionally assigned to each study based on the domain- assessments. The overall judgment considered the nature and potential impact of concerns across domains rather than the number of domains rated as low, unclear, or high risk. Studies with low risk across all four domains were considered to have low overall risk of bias. Studies with one or more domains rated as unclear because of insufficient reporting were considered to have some concerns regarding overall risk of bias. Particular consideration was given to concerns in the index test and flow and timing domains because these may influence the estimates of diagnostic performance. An unclear rating was not considered equivalent to demonstrated high risk of bias. Disagreements were resolved through discussion.

### Diagnostic value measures

Diagnostic value in the present study was analyzed using proportions of positive findings (sensitivity) of the index tests. As references for the interpretation of sensitivity, the following classification was used: 0 to 0.4, low; 0.4 to 0.7, moderate; and 0.7 to 1.0, high [11].

### Synthesis of results

Analyses were conducted for every index test in comparison to the reference standard (MRI verified FS) and for every index test in comparison to different MRI verified diagnoses such as CSS or DH (when available). The results of patients with FS were compared to corresponding values of index test from a different diagnostic group i.e. CSS or DH. Methodological heterogeneity was assessed for patient age, sex, clinical setting, index tests, and MRI. Due to heterogeneity in study populations and concerns about bias arising from insufficient reporting of clinical test procedures, a meta-analysis was not performed.

### Deviation from protocol

In the original search of this study, the diagnosis MRI verified lumbar lateral recess stenosis was included. No study with patient history nor clinical examination findings that were correlated to the diagnosis was found and thus not included in the study. Diagnostic accuracy was intended to be assessed using sensitivity, specificity, predictive values, and likelihood ratios. However, no studies included in this review consisted of healthy controls or of symptomatic patients without MRI-verified diagnosis such as FS, CSS and DH. Consequently, specificity could not be calculated and thus, studies in the present review were only analyzed using sensitivity (proportion of positive findings of the index test).

## Results

### Study selection

**Fig. 1:**
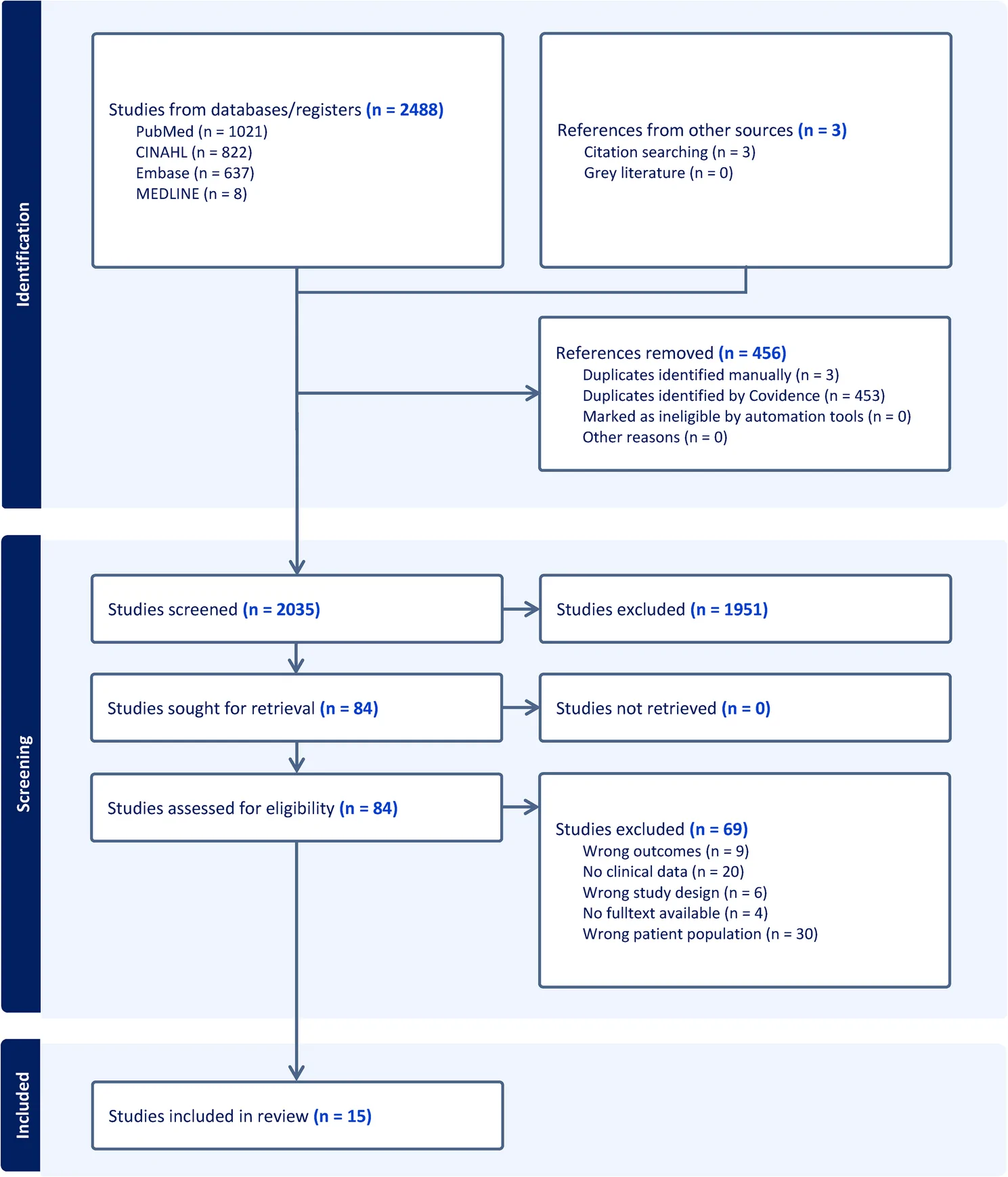
Study flowchart

The study selection process is presented in the PRISMA flow diagram (Fig. 1). A total of 2,488 records were identified through database searching, including PubMed (*n* = 1021), CINAHL (*n* = 822), Embase (*n* = 637) and MEDLINE (*n* = 8). In addition, 3 records were identified through other sources. After removal of duplicates 2,035 records remained for title and abstract screening.

Following title and abstract screening, 1,951 records were excluded and 84 full-text articles were assessed for eligibility. Of these, 61 studies were excluded for the following reasons: wrong patient population (*n* = 30), no clinical data (*n* = 20), wrong outcomes (*n* = 9), wrong study design (*n* = 6), and no full text available (*n* = 4) (Appendix 3). In total, 15 studies were.

included in the review [6, 7, 12–24]. Of the 2,488 studies, 416 were identified in the additional search from October 2024 to September 2026. However, no new studies were included.

### Study characteristics

The 15 included studies comprised mainly cohort and cross-sectional designs [6, 7, 12–24]. The studies included a total of 1,439 participants (Table 1). All except one study [12] were conducted in hospital settings, with most populations consisting of surgical or pre-surgical patients. Sample sizes varied substantially (*n* = 17–300), and the mean age of participants was generally between 60 and 72 years, although some studies included younger populations [7, 12]. The proportion of male and female participants varied and was reported in some studies (Table 1).

**Table 1:** Demographics and characteristics of included studies

Study	Study design; Inclusion; Setting	Population characteristics	Clinical characteristics	Index test	Reference standard
Dobbs et al., 2016 [12]	Cohort; consecutive recruitment; outpatient clinic	*n* = 30; CSS n 14; FS n 16; (64 years; 57% male)	Levels of MRI verified FS: L4-S1 VAS/Disability: NR	**Clinical test**: Lumbar extension test (LE)	MRI according to Lee system
Doi et al., 2011[13]	Cross-sectional; consecutive recruitment; hospital	*n* = 17; FS; n 17; pre-surgical decompression; (68 years; 41% male)	Levels of MRI verified FS: L3-4 *n* = 4, L4-5 *n* = 2, L5-S1 *n* = 11; VAS/Disability NR	**Clinical test**: SLR; LE Sensory deficit; Motor weakness	MRI according to Lee system; CT
Eguchi et al., 2017 [14]	Cohort; consecutive recruitment; hospital	*n* = 43; FS *n* = 13 (72 years); CSS *n* = 30 (73 years); sex NR	Levels of MRI verified FS: L3-4 *n* = 2, L4-5 *n* = 2, L5-S1 *n* = 9; VAS leg pain 80	**Patient history**: Walking distance < 500 m	MRI according to Lee system
Ekedahl et al., 2018 [6]	Prospective cohort; consecutive recruitment; hospital	*n* = 52; DH assessed as extrusion *n* = 27 (56 years; 12% male); FS *n* = 25 (61 years; 15% male)	Levels of MRI verified FS: L2-4 *n* = 6, L4-5 *n* = 19; VAS leg pain 43; Disability 39	**Clinical test**: SLR; Slump test; Sensory deficits; Motor weakness and Reflex impairment	MRI according to Lee system
Heard et al., 2023 [15]	Prospective cohort; hospital	*n* = 121; FS n 121; (59 years; 35% male)	Levels of MRI verified FS: L1-3 *n* = 17 L3-4 *n* = 15, L4-5 *n* = 49 L5-S1 *n* = 30; VAS leg pain 65; Disability 43	Clinical test: Motor weakness	MRI according to Lee system
Jenis et al., 2001 [16]	Retrospective cohort; hospital	*n* = 65; FS; (51 years; 49% male)	Levels of MRI verified FS: NR; VAS/Disability NR	**Clinical test**: SLR; LE; Sensory deficits, Motor weakness and Reflex impairment	MRI according to Lee system; CT
Jönsson et al., 1993 [7]	Cross-sectional; prospective consecutive recruitment; hospital	*n* = 300; DH assessed as complete or incomplete; *n* = 100 (43 years; 65% male); FS *n* = 100 (41 years; 32% male); CSS *n* = 100 (65 years; 54% male)	Levels of MRI verified FS: L3-4 *n* = 10, L4-S1 *n* = 90; VAS/Disability NR	**Patient history**: Walking distance < 500 m; Leg pain at rest **Clinical test**: SLR; LE; Sensory deficits, Motor weakness and Reflex impairment	MRI not specified system; CT; myelography
Konno et al., 2007 [17]	Cross-sectional; surgical series; hospital	*n* = 137; CSS n 50, FS n 87; (71 years; 47% male)	Levels of MRI verified FS: NR; VAS/DisabilityNR	**Clinical test**: SLR; LE; Sensory deficits, Motor weakness and Reflex impairment	MRI not specified system
Lee et al., 2024 [18]	Retrospective cohort; hospital	*n* = 186; FS *n* = 55 (68 years; 29% male); CSS *n* = 131 (65 years; 40% male)	Levels of MRI verified FS: L4-S1; VAS leg pain 62; Disability 47	**Clinical test**: Sensory deficit; Motor weakness	MRI according to Lee system
Ohba et al., 2015 [19]	Cohort; consecutive; hospital	*n* = 68; FS *n* = 29 (72 years; 45% male); CSS *n* = 39 (72 years; 67% male)	Levels of MRI verified FS: L5-S1 *n* = 68; VAS/Disability NR	**Patient history**: Leg pain at rest **Clinical test**: SLR; LE	MRI according to Lee system
Takeuchi et al., 2015 [20]	Prospective cohort; hospital	*n* = 61; FS n 61; (60 years; 57% male)	Levels of MRI verified FS: L4-S1 *n* = 61; VAS leg pain 74	**Clinical test**: Motor weakness; LE	MRI according to Lee system
Watanabe et al., 2011 [21]	Retrospective cohort; hospital	*n* = 31; FS n 31; (61 years; 71% male)	Levels of MRI verified FS: L3-S1 *n* = 31; VAS/Disability NR	**Patient history**: Walking distance < 500 m; Leg pain at rest **Clinical test**: SLR; LE; Motor weakness	MRI according to Lee system
Yamada et al., 2014 [23]	Cross-sectional; prospective; hospital	*n* = 98; FS *n* = 38 (69 years; 55% male); CSS *n* = 60 (71 years; 57% male)	Levels of MRI verified FS: L5-S1 *n* = 98; VAS leg pain 66	**Clinical test**: Motor weakness; SLR; LE	MRI according to Lee system
Yamada et al., 2015 [22]	Prospective cohort; hospital	*n* = 100; FS *n* = 49 (69 years; 55% male); CSS *n* = 51 (65 years; 47% male)	Levels of MRI verified FS: L5-S1 *n* = 49; VAS/Disability NR	**Patient history**: Walking distance < 500 m; **Clinical test**: SLR; LE; Sensory deficit; Motor weakness; Bonnet test and Freiburg test	MRI according to Lee system
Yamada et al., 2023 [24]	Cohort; hospital	*n* = 30; FS n 30; with nerve root edema *n* = 20 (71 years); without nerve root edema *n* = 10 (67 years) (43% male)	Levels of MRI verified FS: L5-S1 *n* = 30; VAS leg pain 55	**Patient history**: Walking distance < 500 m; Leg pain at rest **Clinical test**: SLR; LE; Motor weakness	MRI according to Lee system

Abbreviations: FS = foraminal stenosis; CSS = central stenosis; DH = disc herniation; LE = lumbar extension test; SLR = straight leg raise test; NR = not reported. Mean age and percentages of male participants, in brackets, are rounded to the nearest whole number

### Index tests and reference standard methods assessed

Index tests were categorized into patient history variables and clinical examination findings. Patient history variables were reported in 6 studies and included Walking distance < 500 m and Leg pain at rest [7, 14, 19, 21, 24] (Table 1).

Clinical examination tests were more frequently reported and included the straight leg raise test (SLR; *n* = 10), Lumbar extension test (LE; *n* = 11) [6, 7, 12, 13, 16, 17, 19–24] and Neurological deficits such as Sensory deficit, Motor weakness and Reflex impairment (*n* = 12) [6, 7, 13, 17, 18, 21–23]. The three remaining tests were Bonnet’s test, Freiberg’s test and Slump test.

Lumbar extension test (LE) was slightly differently described and performed in the included studies. The Kemp test is a lumbar extension test commonly performed in extension and ipsilateral lumbar rotation. Other lumbar extension tests are performed without rotation. In this review, all tests where a lumbar extension pain provocation test was performed, the test was regarded as LE.

MRI-verified FS was used as the reference standard in all studies, either alone or in 3 studies in combination with computer tomography (CT), myelography or CT discography [7, 13, 16]. The comparison groups consisted of patients with MRI-verified CSS or DH (Table 1).

### Risk of bias and applicability

The risk of bias and applicability concerns assessed using QUADAS-2 are presented in Table 2. Overall risk of bias was judged as low in two studies [6, 12], while the remaining studies had some concerns. These concerns were mainly related to insufficient reporting in the index test and flow and timing domains rather than clear evidence of methodological flaws.

**Table 2:** Risk of bias and applicability concerns according to the QUADAS-2 assessment

Study	Risk of bias				Overall risk of bias	Applicability concerns		
	Patient selection	Index test	Reference standard	Flow & timing		Patient selection	Index test	Reference standard
Dobbs et al., 2016 [12]	Low	Low	Low	Low	**Low**	Low	Low	Low
Doi et al., 2011 [13]	Low	Unclear	Low	Unclear	**Some concerns**	Low	Low	Low
Eguchi et al., 2017[14]	Low	Low	Low	Unclear	**Some concerns**	Low	Low	Low
Ekedahl et al., 2018 [6]	Low	Low	Low	Low	**Low**	Low	Low	Low
Heard et al., 2023 [15]	Low	Unclear	Low	Unclear	**Some concerns**	Low	Low	Low
Jenis et al., 2001 [16]	Low	Unclear	Unclear	Unclear	**Some concerns**	Low	Unclear	Unclear
Jönsson et al., 1993 [7]	Low	Unclear	Unclear	Unclear	**Some concerns**	Low	Unclear	Unclear
Konno et al., 2007 [17]	Low	Low	Low	Unclear	**Some concerns**	Low	Low	Low
Lee et al., 2024 [18]	Unclear	Unclear	Unclear	Unclear	**Some concerns**	Unclear	Unclear	Low
Ohba et al., 2015 [19]	Low	Unclear	Low	Unclear	**Some concerns**	Low	Unclear	Low
Takeuchi et al., 2015 [20]	Low	Low	Low	Unclear	**Some concerns**	Low	Low	Low
Watanabe et al., 2011 [21]	Low	Unclear	Low	Unclear	**Some concerns**	Low	Low	Low
Yamada et al., 2014 [23]	Low	Low	Low	Unclear	**Some concerns**	Low	Low	Low
Yamada et al., 2015 [22]	Low	Low	Low	Unclear	**Some concerns**	Low	Low	Low
Yamada et al., 2023 [24]	Low	Unclear	Low	Unclear	**Some concerns**	Low	Low	Low

**Risk-of-bias ratings**: Low = low risk of bias; Unclear = insufficient reporting to determine risk; High = high risk of bias. Overall risk of bias: Low = low risk across all domains; Some concerns = one or more domains unclear without evidence of demonstrated high risk. QUADAS-2 = Quality Assessment of Diagnostic Accuracy Studies-2

The index test domain was judged as unclear in seven studies [7, 13, 15, 16, 19, 21, 24], primarily because of insufficient reporting regarding blinding or the procedures used to perform and interpret the index tests. The flow and timing domain was judged as unclear in nine studies [13–15, 17, 19–24], mainly because the interval between the index test and reference standard, or the flow of participants through the study, was insufficiently reported.

Applicability concerns were generally low. However, unclear applicability concerns were identified in three studies [7, 16, 18], particularly in relation to the index test and reference standard.

### Diagnostic value of patient history

Two patient history measures were found. For Walking distance < 500 m and Pain at rest, the proportion of positive findings ranged from 40% to 85% and from 46% to 86%, respectively (Tables 3 and 4).

**Table 3:** Proportion of positive index test (Walking distance < 500 m) findings in relation to MRI-verified foraminal stenosis (number and %)

Study	Diagnosis	Pos/total	%
Eguchi 2017[14]	FS	11/13	85
Jönsson 1993[7]	FS	40/100	40
Watanabe2011[21]	FS	23/31	74
Yamada 2015[22]	FS	34/49	69

Abbreviations: FS = foraminal stenosis; Pos = positive findings

**Table 4:** Proportion of positive index test (Pain at rest) findings in relation to MRI-verified foraminal stenosis

Study	Diagnosis	Pos/total	%
Jönsson 1993[7]	FS	86/100	86
Ohba 2015[19]	FS	14/29	48
Watanabe2011[21]	FS	13/28	46
Yamada 2015[22]	FS	43/49	88
Yamada 2023[24]	FS	23/30	77

Abbreviations: FS = foraminal stenosis; Pos = positive findings

### Diagnostic value of clinical examination test

Eight clinical examination tests were found. Three of those were less frequently used and were evaluated in two studies [6, 22]. The tests were Bonnet test (proportion of positive findings 65%) [22], Freiberg test (proportion of positive findings 45%) [22] and Slump test (proportion of positive findings 48%) [6]. For the remaining five clinical examination tests, the proportion of positive findings ranged from 9% to 88% (Tables 5, 6, 7, 8 and 9). The highest was reported for Sensory deficit [13] (Table 7) and the lowest for Motor weakness [17] (Table 8).

**Table 5:** Proportion of positive index test (Straight leg raise) findings in relation to MRI-verified foraminal stenosis (number and %)

Study	Diagnosis	Pos/total	%
Doi 2011[13]	FS	11/17	65
Ekedahl 2018[6]	FS	8/25	32
Jenis 2001[16]	FS	27/65	42
Jönsson 1993[7]	FS	51/100	51
Konno 2007[17]	FS	19/87	22
Ohba 2015[19]	FS	7/29	24
Watanabe2011[21]	FS	10/31	32
Yamada 2014[23]	FS	7/38	18
Yamada 2015[22]	FS	7/49	14
Yamada 2023[24]	FS	10/30	33

Abbreviations: FS = foraminal stenosis; Pos = positive findings

**Table 6:** Proportion of positive index test (Lumbar extension) findings in relation to MRI-verified foraminal stenosis

Study	Diagnosis	Pos/total	%
Dobbs 2016[12]	FS	13/16	81
Doi 2011[13]	FS	13/17	76
Jenis 2001[16]	FS	37/65	57
Jönsson 1993[7]	FS	72/100	72
Konno 2007[17]	FS	55/87	63
Ohba 2015[19]	FS	12/29	41
Takeuchi 2015[20]	FS	33/61	54
Watanabe2011[21]	FS	15/19	79
Yamada 2014[23]	FS	26/38	68
Yamada 2015[22]	FS	39/49	80
Yamada 2023[24]	FS	19/30	63

Abbreviations: FS = foraminal stenosis; Pos = positive findings

**Table 7:** Proportion of positive index test (Sensory deficit) findings in relation to MRI-verified foraminal stenosis

Study	Diagnosis	Pos/total	%
Doi 2011[13]	FS	15/17	88
Ekedahl 2018[6]	FS	14/25	56
Jenis 2001[16]	FS	21/65	32
Jönsson 1993[7]	FS	54/100	54
Konno 2007[17]	FS	43/87	49
Lee 2024[18]	FS	11/55	20
Yamada 2015[22]	FS	29/49	59

Abbreviations: FS = foraminal stenosis; Pos = positive findings

**Table 8:** Proportion of positive index test (Motor weakness) findings in relation to MRI-verified foraminal stenosis (number and %)

Study	Diagnosis	Pos/total	%
Doi 2011[13]	FS	10/17	59
Ekedahl 2018[6]	FS	11/25	44
Heard 2023[15]	FS	67/121	55
Jenis 2001[16]	FS	31/65	48
Jönsson 1993[7]	FS	55/100	55
Konno 2007[17]	FS	8/87	9
Lee 2024[18]	FS	12/55	22
Takeuchi 2015[20]	FS	9/61	15
Watanabe2011[21]	FS	10/31	32
Yamada 2014[23]	FS	11/38	29
Yamada 2015[22]	FS	12/49	24
Yamada 2023[24]	FS	6/30	20

Abbreviations: FS = foraminal stenosis; Pos = positive findings

**Table 9:** Proportion of positive index test (Reflex impairment) findings in relation to MRI-verified foraminal stenosis (number and %)

Study	Diagnosis	Pos/total	%
Ekedahl 2018[6]	FS	9/25	36
Jenis 2001[16]	FS	15/65	23
Jönsson 1993[7]	FS	37/100	37
Konno 2007[17]	FS	24/87	28

Abbreviations: FS = foraminal stenosis; Pos = positive findings

### Synthesis of results

Two patient history measures and five clinical examination tests were reported in more than one study and the results were summarized for each index test (Table 10). The index tests with highest average proportion of positive findings in detecting FS were leg pain at rest (73%) and lumbar extensions test (LE; 66%); and the tests with the lowest average proportion of positive findings were SLR (33%) and Reflex impairment (33%) (Table 10).

**Table 10:** Comparison of average proportion of positive findings in index tests in relation to MRI diagnoses (number and %)

		Foraminal stenosis	Central spinal stenosis	Disc herniation	
Index item					Total
SLR Pos		156	75	104	335
SLR Neg		314	225	23	563
SLR % Pos		33	25	82	
Sensory deficit Pos		187	137	81	405
Sensory deficit Neg		211	196	46	453
Sensory deficit % Pos		47	41	64	
Motor weakness Pos		177	117	62	356
Motor weakness Neg		385	275	65	725
Motor weakness % Pos		38	30	49	
Reflex impairment Pos		92	35	10	137
Reflex impairment Neg		186	15	17	218
Reflex impairment % Pos		33	70	37	
Lumbar extension Pos		321	197	74	592
Lumbar extension Neg		167	102	26	295
Lumbar extension % Pos		66	66	74	
Pain at rest Yes		136	63	85	284
Pain at rest No		51	37	15	103
Pain at rest % Yes		72	63	85	
Walking distance < 500 m		131	144	111	386
Walking distance > 500 m		92	7	19	118
Walking distance % <500 m		59	95	85	

Abbreviations: Pos = positive findings; Neg = negative findings

In comparison between the FS group and the DH group regarding SLR, Sensory deficit and Walking distance < 500 m, the proportions of positive findings were more common in the DH group. In comparison between the FS group and the CSS group, the proportions of positive findings were higher for the CSS group regarding the Walking distance < 500 m and Reflex impairment; by contrast, the proportion of findings was higher for the Leg pain at rest measure in the FS group compared to the CSS group (Table 10).

## Discussion

### Summary of evidence

The diagnosis of lumbar foraminal stenosis (FS) remains a subject of ongoing debate [25]. The diagnosis is typically based on a combination of patient history and careful clinical examination, with magnetic resonance imaging (MRI) used to support or confirm the clinical hypothesis [25]. The objectives of these assessments often shift based on the intended therapy. Furthermore, clinical guidelines for low back pain suggest for the judicious use of imaging, prioritized according to the patient’s level of disability [26]. The patient history and clinical examination are indisputably central to the diagnostic process, and the findings of this study carry significant clinical weight [25]. No previous systematic reviews with meta-analysis have been conducted on this topic. Our aim was to determine the diagnostic accuracy of patient history and clinical examination tests for diagnosing lumbar radicular pain due to FS. Diagnostic accuracy reflects upon both sensitivity of a test and specificity. A test’s ability to correctly identify a diagnosis (sensitivity) is crucial, however, specificity cannot be neglected. High specificity indicates high proportion of patients with negative MRI-findings who are correctly tested with a negative index test. Unfortunately, specificity of the index test in diagnosing FS could not be calculated. This study is limited to the sensitivity (proportions of positive findings) in diagnosing lumbar FS.

### Reference standard

The reference standard in this study was MRI verified FS according to Lee system [9], where grades II and III are considered as high-grade nerve compression and verify the diagnosis of FS. This examination is well used, and it is regarded as both valid and reliable [27, 28]. A limitation of the system is that MRI-verified lumbar FS may be present without any symptoms, particularly in elderly patients. However, the patients included in the present studies constituted a more homogeneous group, consisting mainly of surgical candidates with an established diagnosis. The other two MRI-verified diagnoses in our study, namely CSS and DH, were not specifically described but also consisted primarily of surgical candidates.

### Study population

The included studies primarily involved surgical candidates recruited from hospital settings, with limited variation in age, disability severity, and leg pain intensity. Positive clinical test findings are likely more common in this population than in an unselected primary care population [4]. Although the study group may be relatively homogeneous, it also means that the result cannot be generalized to all subjects with FS. Further studies are needed on a broader patient group with lumbar radicular symptoms with and without MRI verified diagnoses.

### Index tests

All index tests in this study are dichotomous variables. To include continuous variables that were dichotomized using pre-set cut-off points could have been an adequate option. However, no such variables were found in the search.

Most of the tests included in this study have previously been tested with adequate reliability. Two of the tests are rarely tested in clinical trials, Bonnet and Freiberg test [29]. Those tests showed moderate ability to detect FS [23]. After the tests have been tested with adequate reliability, they might be valuable contributions in future trials and clinical practice. The lumbar extension test in our study was a collection of slightly different tests. The Kemp test was the most commonly used test and has previously demonstrated high reliability [12]. However, the reliability of the other lumbar extension tests remains uncertain and may influence our result.

Prior studies of neurodynamic tests such as SLR in detecting nerve compression caused by DH reported high sensitivity for SLR [4]. These findings are consistent with our results of patients with DH. Interestingly, the proportion of positive SLR findings for detecting FS was significantly lower. These results may be explained by limitations of the included studies, such as variation in the level of nerve root involvement. This may be explained by a higher frequency of femoral nerve involvement; however, the affected levels reported in the present studies were predominantly the L4 and L5 nerve roots. (Table 1). Individual neurologic tests have previously been presented with insufficient sensitivity in detecting radicular symptoms caused by DH [4] which also is in accordance with our results regarding both DH and FS.

### Risk of bias

We assessed risk of bias using the QUADAS-2 tool. Overall risk of bias was judged as low in two studies, while the remaining studies had some concerns, mainly due to insufficient reporting in the index test and flow and timing domains. In particular, several studies provided insufficient information about blinding and the timing between the clinical assessment and MRI. Consequently, it was not always possible to determine whether the clinical assessments were performed independently of the MRI findings or whether the time interval between assessments could have influenced the results. Importantly, these unclear judgments primarily reflect limitations in reporting rather than evidence of methodological bias. Nevertheless, the uncertainty in these domains reduces confidence in the estimates and warrants cautious interpretation of the findings.

### Clinical aspect

No single clinical index test demonstrated sufficiently diagnostic value to support use as a stand-alone test to detect lumbar FS. However, unilateral leg pain and Leg pain at rest seem to indicate DH or FS rather than CSS. Intense unilateral radiating pain, particularly in the S1 dermatome, a positive SLR (or Slump test), and an impaired Achilles reflex indicate S1 radiculopathy due to DH rather than FS. From a primary care perspective, the presence of a positive SLR and severe pain persisting without improvement for 6–8 weeks indicates the need for referral for MRI [2]. However, patients with lumbar FS are often assessed with a negative SLR, which increases the risk of a missed diagnosis. In secondary care, the clinical assessment may have a different focus. Here, a combination of variables may improve the diagnostic procedure of FS and verify the level of radicular pain, typically including a unilateral sensory deficit, motor weakness corresponding to the same nerve root, and a positive lumbar extension test [25]. This approach, together with a positive selective nerve root block, constitutes the current standard procedure [30, 31]. In summary, our results do not contradict the standard procedure but suggest placing less emphasis on some index tests e.g. SLR test.

### Limitations and strength

There are always limitations to such a study. One limitation of this study is that the gold standard has been questioned. MRI as a reference test can be challenged as FS is frequently seen on MR images in symptom-free persons. However, the agreement between radiologists and spine surgeons for preoperative MRI findings of lumbar FS is high [27, 28].

Another limitation is that no healthy controls or symptomatic patients without MRI-verified diagnosis were included in the present study, thus the diagnostic accuracy analysis was limited to sensitivity. Furthermore, most of the studies included in this review involve patients who are candidates for surgery. Further studies are needed in primary care populations with lumbar FS and should also include control groups. The diagnoses of lumbar lateral recess stenosis and spondylolistheses may also play an important role in cases of radicular pain. Unfortunately, we found no studies where patients with these diagnoses were included.

A major strength of this study is that it synthesizes the current evidence on the diagnostic value of patient history and clinical examination tests for detecting FS and compares these findings with those reported for similar diagnoses. Our results support the current standard procedure while suggesting that less emphasis should be placed on the SLR test. Further studies are needed to evaluate the accuracy of clinical tests for diagnosing FS, including analyses of both sensitivity and specificity.

## Conclusions

This systematic review concluded that SLR has the lowest ability to detect lumbar foraminal stenosis, whereas positive Lumbar extension test and Leg pain at rest have the highest ability to detect the diagnosis. These findings should be interpreted cautiously given methodological uncertainties and limited diagnostic accuracy evidence.

## Supplementary Information

Below is the link to the electronic supplementary material.

Supplementary Material 1

Supplementary Material 2

Supplementary Material 3

## Data Availability

The datasets used and/or analyzed during the current study are available from the corresponding author on reasonable request.

## Abbreviations

CSS: Central spinal stenosis
DH: Disc herniation
FS: Foraminal stenosis
LBP: Low back pain
LE: lumbar extension test
MRI: magnetic resonance imaging
PRISMA: DTA–Preferred Reporting Items for Systematic Reviews and Meta–Analysis Protocols for diagnostic test accuracy studies
Quality Assessment of Diagnostic Accuracy Studies: 2 (QUADAS 2)
SLR: Straight leg raise

## Abbreviations

CSS: Central spinal stenosis
DH: Disc herniation
FS: Foraminal stenosis
LBP: Low back pain
LE: lumbar extension test
MRI: magnetic resonance imaging
PRISMA: DTA–Preferred Reporting Items for Systematic Reviews and Meta–Analysis Protocols for diagnostic test accuracy studies
Quality Assessment of Diagnostic Accuracy Studies: 2 (QUADAS 2)
SLR: Straight leg raise

## References

[1] Ishimoto Y, Yoshimura N, Muraki S, Yamada H, Nagata K, Hashizume H, Takiguchi N, Minamide A, Oka H, Kawaguchi H, et al. Associations between radiographic lumbar spinal stenosis and clinical symptoms in the general population: the Wakayama Spine Study. Osteoarthritis Cartilage. 2013;21(6):783–8. 10.1016/j.joca.2013.02.656 23473979

[2] George SZ, Fritz JM, Silfies SP, Schneider MJ, Beneciuk JM, Lentz TA, Gilliam JR, Hendren S, Norman KS. Interventions for the Management of Acute and Chronic Low Back Pain: Revision 2021. J Orthop Sports Phys Ther. 2021;51(11):Cpg1–60. 10.2519/jospt.2021.0304 PMC10508241 34719942

[3] Pojskic M, Bisson E, Oertel J, Takami T, Zygourakis C, Costa F. Lumbar disc herniation: Epidemiology, clinical and radiologic diagnosis WFNS spine committee recommendations. World Neurosurgery: X. 2024;22:100279. 10.1016/j.wnsx.2024.100279 PMC10911853 38440379

[4] van der Windt DA, Simons E, Riphagen II, Ammendolia C, Verhagen AP, Laslett M, Devillé W, Deyo RA, Bouter LM, de Vet HC et al. Physical examination for lumbar radiculopathy due to disc herniation in patients with low-back pain. Cochrane Database Syst Rev 2010(2):Cd007431.10.1002/14651858.CD007431.pub220166095

[5] Cook CJ, Cook CE, Reiman MP, Joshi AB, Richardson W, Garcia AN. Systematic review of diagnostic accuracy of patient history, clinical findings, and physical tests in the diagnosis of lumbar spinal stenosis. Eur Spine J. 2020;29(1):93–112. 10.1007/s00586-019-06048-4 31312914

[6] Ekedahl H, Jönsson B, Annertz M, Frobell RB. Accuracy of Clinical Tests in Detecting Disk Herniation and Nerve Root Compression in Subjects With Lumbar Radicular Symptoms. Arch Phys Med Rehabil. 2018;99(4):726–35. 10.1016/j.apmr.2017.11.006 29253501

[7] Jönsson B, Strömqvist B. Symptoms and signs in degeneration of the lumbar spine. A prospective, consecutive study of 300 operated patients. J Bone Joint Surg Br. 1993;75(3):381–5. 10.1302/0301-620X.75B3.8496204 8496204

[8] McInnes MDF, Moher D, Thombs BD, McGrath TA, Bossuyt PM, Clifford T, Cohen JF, Deeks JJ, Gatsonis C, Hooft L, et al. Preferred Reporting Items for a Systematic Review and Meta-analysis of Diagnostic Test Accuracy Studies: The PRISMA-DTA Statement. JAMA. 2018;319(4):388–96. 10.1001/jama.2017.19163 29362800

[9] Lee S, Lee JW, Yeom JS, Kim KJ, Kim HJ, Chung SK, Kang HS. A practical MRI grading system for lumbar foraminal stenosis. AJR Am J Roentgenol. 2010;194(4):1095–8. 10.2214/AJR.09.2772 20308517

[10] Whiting PF, Rutjes AW, Westwood ME, Mallett S, Deeks JJ, Reitsma JB, Leeflang MM, Sterne JA, Bossuyt PM. QUADAS-2: a revised tool for the quality assessment of diagnostic accuracy studies. Ann Intern Med. 2011;155(8):529–36. 10.7326/0003-4819-155-8-201110180-00009 22007046

[11] Jaeschke R, Guyatt GH, Sackett DL, Guyatt G, Bass E, Brill-Edwards P, Browman G, Cook D, Farkouh M, Gerstein H, et al. Users’ Guides to the Medical Literature: III. How to Use an Article About a Diagnostic Test B. What Are the Results and Will They Help Me in Caring for My Patients? JAMA. 1994;271(9):703–7. 10.1001/jama.271.9.703 8309035

[12] Dobbs R, May S, Hope P. The validity of a clinical test for the diagnosis of lumbar spinal stenosis. Man Therap. 2016;25:27–34. 10.1016/j.math.2016.05.332 27422594

[13] Doi T, Harimaya K, Matsumoto Y, Tono O, Tarukado K, Iwamoto Y. Endoscopic decompression for intraforaminal and extraforaminal nerve root compression. J Orthop Surg Res. 2011;6:16. 10.1186/1749-799X-6-16 PMC3073935 21439083

[14] Eguchi Y, Suzuki M, Yamanaka H, Tamai H, Kobayashi T, Orita S, Yamauchi K, Inage K, Kanamoto H, Abe K, et al. Assessment of Clinical Symptoms in Lumbar Foraminal Stenosis Using the Japanese Orthopaedic Association Back Pain Evaluation Questionnaire. Korean J Spine. 2017;14(1):1–6. 10.14245/kjs.2017.14.1.1 PMC5402862 28407702

[15] Heard JC, Lee Y, Ezeonu T, Lambrechts MJ, Issa TZ, Yalla GR, Tran K, Singh A, Purtill C, Somers S, et al. Does the Severity of Foraminal Stenosis Impact Outcomes of Lumbar Decompression Surgery? World Neurosurg. 2023;179:e296–304. 10.1016/j.wneu.2023.08.081 37633493

[16] Jenis LG, An HS, Gordin R. Foraminal stenosis of the lumbar spine: a review of 65 surgical cases. Am J Orthop (Belle Mead NJ). 2001;30(3):205–11. 11300129

[17] Konno S, Kikuchi S, Tanaka Y, Yamazaki K, Shimada Y, Takei H, Yokoyama T, Okada M, Kokubun S. A diagnostic support tool for lumbar spinal stenosis: a self-administered, self-reported history questionnaire. BMC Musculoskelet Disord. 2007;8:102. 10.1186/1471-2474-8-102 PMC2176057 17967201

[18] Lee HR, Cho JH, Lee DH, Seok SY, Hwang CJ, Lee CS. Exploring the impact of mild-to-moderate foraminal stenosis at L5-S1 on clinical outcomes following L4-5 posterior lumbar interbody fusion. Spine J. 2024;24(5):820–30. 10.1016/j.spinee.2023.12.006 38219839

[19] Ohba T, Ebata S, Fujita K, Sato H, Devin C, Haro H, Devin CJ. Characterization of symptomatic lumbar foraminal stenosis by conventional imaging. Eur Spine J. 2015;24(10):2269–75. 10.1007/s00586-015-3859-4 25772088

[20] Takeuchi M, Wakao N, Kamiya M, Gosho M, Osuka K, Hirasawa A, Niwa A, Aoyama M, Kawaguchi R, Shima H, et al. Diagnostic accuracy of multifidus muscle spontaneous activity by needle electromyography for the detection of lumbar foraminal and lateral exit-zone stenosis. Eur Spine J. 2015;24(10):2281–7. 10.1007/s00586-015-3846-9 25733203

[21] Watanabe K, Yamazaki A, Morita O, Sano A, Katsumi K, Ohashi M. Clinical outcomes of posterior lumbar interbody fusion for lumbar foraminal stenosis: preoperative diagnosis and surgical strategy. J Spinal Disord Tech. 2011;24(3):137–41. 10.1097/BSD.0b013e3181e1cd99 20634730

[22] Yamada H, Oka H, Iwasaki H, Endo T, Kioka M, Ishimoto Y, Nagata K, Takiguchi N, Hashizume H, Minamide A, et al. Development of a support tool for the clinical diagnosis of symptomatic lumbar intra- and/or extra-foraminal stenosis. J Orthop Sci. 2015;20(5):811–7. 10.1007/s00776-015-0743-3 26104220

[23] Yamada K, Aota Y, Higashi T, Ishida K, Nimura T, Konno T, Saito T. Lumbar foraminal stenosis causes leg pain at rest. Eur Spine J. 2014;23(3):504–7. 10.1007/s00586-013-3055-3 PMC3940796 24081690

[24] Yamada K, Aota Y, Saito T, Inaba Y. Correlation between leg pain at rest and spinal nerve edema in symptomatic lumbar foraminal stenosis. Orthop Traumatol Surg Res. 2023;109(2):103119. 10.1016/j.otsr.2021.103119 34666199

[25] Sundaram PS, Regis PAP, Rajadurai OJW, Purushothaman K, Kumar MS, Kanchan N. Assessment of Lumbar Foraminal Stenosis: Diagnostic Value and Clinical Correlation: A Systematic Review. J Orthop Case Rep. 2026;16(3):388–99. 10.13107/jocr.2026.v16.i03.7000 PMC12973068 41815661

[26] O’Connell NE, Cook CE, Wand BM, Ward SP. Clinical guidelines for low back pain: A critical review of consensus and inconsistencies across three major guidelines. Best Pract Res Clin Rheumatol. 2016;30(6):968–80. 10.1016/j.berh.2017.05.001 29103554

[27] Jeong TS, Ahn Y, Lee SG, Kim WK, Son S, Kwon JH. Correlation between MRI Grading System and Surgical Findings for Lumbar Foraminal Stenosis. J Korean Neurosurg Soc. 2017;60(4):465–70. 10.3340/jkns.2016.1010.004 PMC5544375 28689396

[28] Banitalebi H, Espeland A, Anvar M, Hermansen E, Hellum C, Brox JI, Myklebust T, Indrekvam K, Brisby H, Weber C, et al. Reliability of preoperative MRI findings in patients with lumbar spinal stenosis. BMC Musculoskelet Disord. 2022;23(1):51. 10.1186/s12891-021-04949-4 PMC8760672 35033042

[29] Martin HD, Kivlan BR, Palmer IJ, Martin RL. Diagnostic accuracy of clinical tests for sciatic nerve entrapment in the gluteal region. Knee Surg Sports Traumatol Arthrosc. 2014;22(4):882–8. 10.1007/s00167-013-2758-7 24217716

[30] Abe E, Ohara Y, Hara T, Iwamuro H, Nojiri H, Kondo A. Diagnosis of Lumbar Foraminal Stenosis: A Literature Review. J Minim Invasive Spine Surg Tech. 2024;9(Suppl 1):S62–9.

[31] Beynon R, Elwenspoek MMC, Sheppard A, Higgins JN, Kolias AG, Laing RJ, Whiting P, Hollingworth W. The utility of diagnostic selective nerve root blocks in the management of patients with lumbar radiculopathy: a systematic review. BMJ Open. 2019;9(4):e025790. 10.1136/bmjopen-2018-025790 PMC6500311 31005925

