# Supplementary material for: Clinical findings associated with diagnosing lumbar foraminal stenosis: systematic review
Source: BMC Musculoskelet Disord. 2026 Nov 3;27:969. doi: 10.1186/s12891-026-10614-5 (PMC13749674; doi:10.1186/s12891-026-10614-5)
Supplement: Supplementary file 2 [file 12891_2026_10614_MOESM2_ESM.docx]

### **Appendix 2**

### **Databases**

### Date of search: 2024, October 16 and 2026, September 7

### **PubMed (Nationial Library of Medicine)**

Population

#1 (("pain"[MeSH Terms]) OR ("low back pain"[MeSH Terms])) OR ("sciatica"[MeSH Terms])

#2 low back pain[Title/Abstract] OR lumbar pain[Title/Abstract] OR back pain[Title/Abstract] OR lumbar[Title/Abstract] OR sciatic[Title/Abstract] OR sciatica[Title/Abstract] OR LBP[Title/Abstract]

#3 Combination seach #1 OR #2

Target condition

#4 ("spinal stenosis"[MeSH Terms])

#5 foraminal stenosis[Title/Abstract] OR foraminal[Title/Abstract] OR recess stenosis[Title/Abstract] OR lateral stenosis[Title/Abstract] OR extra-foraminal[Title/Abstract] OR extra foraminal[Title/Abstract] OR extraforaminal[Title/Abstract] OR intraforaminal[Title/Abstract] OR intra-foraminal[Title/Abstract] OR root canal stenosis[Title/Abstract] OR lumbar spinal stenosis[Title/Abstract] OR LIEFS[Title/Abstract] OR LSS[Title/Abstract] OR midlumbar nerve root[Title/Abstract] OR lumbar nerve root[Title/Abstract] OR nerve compression[Title/Abstract]

#6 Combination search #4 OR #5

Diagnostic tests-index, clinical/self-reported

#7 ((((("physical examination"[MeSH Terms])) OR ("physical functional performance"[MeSH Terms])) OR ("gait analysis"[MeSH Terms])) OR ("diagnostic techniques, neurological"[MeSH Terms])) OR ("neurologic examination"[MeSH Terms])

#8 clinical examination[Title/Abstract] OR clinical finding*[Title/Abstract] OR physical examination[Title/Abstract] OR neurological[Title/Abstract] OR neurologic[Title/Abstract] OR SLR[Title/Abstract] OR kemp[Title/Abstract] OR sensory deficit[Title/Abstract] OR reflex[Title/Abstract] OR functional test*[Title/Abstract] OR history[Title/Abstract] OR walking[Title/Abstract] OR gait[Title/Abstract] OR assessment[Title/Abstract] OR clinical symptoms[Title/Abstract] OR clinical assessment[Title/Abstract] OR clinical test*[Title/Abstract] OR physical function[Title/Abstract] OR clinical diagnosis[Title/Abstract] OR clinical diagnostic[Title/Abstract] OR diagnostic test*[Title/Abstract] OR diagnostic evaluation[Title/Abstract] OR straight leg rais*[Title/Abstract] OR slump[Title/Abstract] OR straight-leg raise[Title/Abstract] OR straight leg rais*[Title/Abstract] OR sensory test*[Title/Abstract] OR tension test*[Title/Abstract] OR neurodynamic test*[Title/Abstract] OR history items[Title/Abstract] OR Patient-reported[Title/Abstract] OR self-reported[Title/Abstract]

#9 Combination search #7 OR #8

Diagnostic test- MRI

#10 ("magnetic resonance imaging"[MeSH Terms]) OR ("magnetic resonance imaging/methods"[MeSH Terms])

#11 magnetic resonance imaging[Title/Abstract] OR MRI[Title/Abstract] OR MR imaging[Title/Abstract] OR images[Title/Abstract]

#12 Combination search #11 OR #10

Methodological filter- diagnostic accuracy terms

#13 (("predictive value of tests"[MeSH Terms]) OR ("sensitivity and specificity"[MeSH Terms])) OR ("roc curve"[MeSH Terms])

#14 accuracy[Title/Abstract] OR accurately[Title/Abstract] OR diagnostic value[Title/Abstract] OR predictive value[Title/Abstract] OR roc[Title/Abstract] OR likelihood[Title/Abstract] OR sensitivity[Title/Abstract] OR valid*[Title/Abstract] OR predict*[Title/Abstract] OR clinical diagnostic[Title/Abstract] OR receiver operating characteristic[Title/Abstract] OR receiver-operating characteristic[Title/Abstract] OR odds ratio[Title/Abstract] OR dor[Title/Abstract] OR correlat*[Title/Abstract] OR compar*[Title/Abstract] OR relat*[Title/Abstract] OR associat*[Title/Abstract] OR DTA[Title/Abstract]

#15 Combination search #13 OR #14

Combination search Population, target, diagnostic tests, methodological filter with AND

#16 Combination search #3 AND #6 AND #9 AND #12 AND #15

**Embase.com (Elsevier, 1947-present) and MEDLINE**

Population

#1 'pain'/exp OR 'low back pain'/exp OR 'sciatica'/exp

#2 'low back pain':ab,ti OR 'lumbar pain':ab,ti OR 'back pain':ab,ti OR lumbar:ab,ti OR sciatic:ab,ti OR sciatica:ab,ti OR lbp:ab,ti

#3 Combination search #1 OR #2

Target condition

#4 'vertebral canal stenosis'/exp

#5 'foraminal stenosis':ab,ti OR foraminal:ab,ti OR 'recess stenosis':ab,ti OR 'lateral stenosis':ab,ti OR 'extra foraminal':ab,ti OR extraforaminal:ab,ti OR intraforaminal:ab,ti OR 'intra foraminal':ab,ti OR 'root canal stenosis':ab,ti OR 'lumbar spinal stenosis':ab,ti OR liefs:ab,ti OR lss:ab,ti OR 'midlumbar nerve root':ab,ti OR 'lumbar nerve root':ab,ti OR 'nerve compression':ab,ti

#6 Combination search #4 OR #5

Diagnostic tests-index, clinical/self-reported

#7 'physical examination'/exp OR 'physical performance'/exp OR 'gait'/exp OR 'neurologic examination'/exp

#8 'clinical examination':ab,ti OR 'clinical finding*':ab,ti OR 'physical examination':ab,ti OR neurological:ab,ti OR neurologic:ab,ti OR slr:ab,ti OR kemp:ab,ti OR 'sensory deficit':ab,ti OR reflex:ab,ti OR 'functional test*':ab,ti OR history:ab,ti OR walking:ab,ti OR gait:ab,ti OR assessment:ab,ti OR 'clinical symptoms':ab,ti OR 'clinical assessment':ab,ti OR 'clinical test*':ab,ti OR 'physical function':ab,ti OR 'clinical diagnosis':ab,ti OR 'clinical diagnostic':ab,ti OR 'diagnostic test*':ab,ti OR 'diagnostic evaluation':ab,ti OR slump:ab,ti OR 'straight-leg raise':ab,ti OR 'straight leg rais*':ab,ti OR 'sensory test*':ab,ti OR 'tension test*':ab,ti OR 'neurodynamic test*':ab,ti OR 'history items':ab,ti OR 'patient reported':ab,ti OR 'self reported':ab,ti

#9 Combination search #7 OR #8

Diagnostic test- MRI

#10 'nuclear magnetic resonance imaging'/exp

#11 'magnetic resonance imaging':ab,ti OR mri:ab,ti OR 'mr imaging':ab,ti OR images:ab,ti

#12 Combination search #10 OR #11

Methodological filter- diagnostic accuracy terms

#13 ('predictive value'/exp OR 'sensitivity'/exp) AND 'specificity'/exp OR 'receiver operating characteristic'/exp

#14 accuracy:ab,ti OR accurately:ab,ti OR 'diagnostic value':ab,ti OR 'predictive value':ab,ti OR roc:ab,ti OR likelihood:ab,ti OR sensitivity:ab,ti OR valid*:ab,ti OR predict*:ab,ti OR 'clinical diagnostic':ab,ti OR 'receiver operating characteristic':ab,ti OR 'receiver-operating characteristic':ab,ti OR 'odds ratio':ab,ti OR dor:ab,ti OR correlat*:ab,ti OR compar*:ab,ti OR relat*:ab,ti OR associat*:ab,ti OR dta:ab,ti

#15 Combination search #13 OR #14

Combination search Population, target, diagnostic tests, methodological filter with AND

#16 Combination search #3 AND #6 AND #9 AND #12 AND #15

#17 #16 AND [embase]/lim NOT ([embase]/lim AND [medline]/lim)

#17 NOT ‘conference abstract’:it

**Cinahl Complete**

Population

#1 (MH "Pain+") OR (MH "Low Back Pain") OR (MH "Sciatica")

#2 TI ( low back pain OR lumbar pain OR back pain OR lumbar OR sciatic OR sciatica OR LBP ) OR AB ( low back pain OR lumbar pain OR back pain OR lumbar OR sciatic OR sciatica OR LBP )

#3 Combination search #1 OR #2

Target condition

#4 (MH "Spinal Stenosis")

#5 TI ( foraminal stenosis OR foraminal OR recess stenosis OR lateral stenosis OR extra-foraminal OR extra foraminal OR extraforaminal OR intraforaminal OR intra-foraminal OR root canal stenosis OR lumbar spinal stenosis OR LIEFS OR LSS OR midlumbar nerve root OR lumbar nerve root OR nerve compression ) OR AB ( foraminal stenosis OR foraminal OR recess stenosis OR lateral stenosis OR extra-foraminal OR extra foraminal OR extraforaminal OR intraforaminal OR intra-foraminal OR root canal stenosis OR lumbar spinal stenosis OR LIEFS OR LSS OR midlumbar nerve root OR lumbar nerve root OR nerve compression )

#6 Combination search #4 OR #5

Diagnostic tests-index, clinical/self-reported

#7 (MH "Physical Examination+") OR (MM "Functional Status") OR (MH "Gait Analysis") OR (MH "Neurologic Examination+")

#8 TI ( clinical examination OR clinical finding* OR physical examination OR neurological OR neurologic OR SLR OR kemp OR sensory deficit OR reflex OR functional test* OR history OR walking OR gait OR assessment OR clinical symptoms OR clinical assessment OR clinical test* OR physical function OR clinical diagnosis OR clinical diagnostic OR diagnostic test* OR diagnostic evaluation OR straight leg rais* OR slump OR straight-leg raise OR straight leg rais* OR sensory test* OR tension test* OR neurodynamic test* OR history items OR Patient-reported OR self-reported ) OR AB ( clinical examination OR clinical finding* OR physical examination OR neurological OR neurologic OR SLR OR kemp OR sensory deficit OR reflex OR functional test* OR history OR walking OR gait OR assessment OR clinical symptoms OR clinical assessment OR clinical test* OR physical function OR clinical diagnosis OR clinical diagnostic OR diagnostic test* OR diagnostic evaluation OR straight leg rais* OR slump OR straight-leg raise OR straight leg rais* OR sensory test* OR tension test* OR neurodynamic test* OR history items OR Patient-reported OR self-reported )

#9 Combination search #7 OR #8

Diagnostic test- MRI

#10 (MH "Magnetic Resonance Imaging+")

#11 TI ( magnetic resonance imaging OR MRI OR MR imaging OR images ) OR AB ( magnetic resonance imaging OR MRI OR MR imaging OR images )

#12 Combination search #10 OR #11

Methodological filter- diagnostic accuracy terms]

#13 (MH "Predictive Value of Tests") OR (MH "Sensitivity and Specificity") OR (MH "ROC Curve")

#14 TI ( accuracy OR accurately OR diagnostic value OR predictive value OR roc OR likelihood OR sensitivity OR valid* OR predict* OR clinical diagnostic OR receiver operating characteristic OR receiver-operating characteristic OR odds ratio OR dor OR correlat* OR compar* OR relat* OR associat* OR DTA ) OR AB ( accuracy OR accurately OR diagnostic value OR predictive value OR roc OR likelihood OR sensitivity OR valid* OR predict* OR clinical diagnostic OR receiver operating characteristic OR receiver-operating characteristic OR odds ratio OR dor OR correlat* OR compar* OR relat* OR associat* OR DTA )

#15 Combination search #13 OR #14

Combination search Population, target, diagnostic tests, methodological filter with AND

#16 Combination search #3 AND #6 AND #9 AND #12 AND #15
