# Supplementary material for: Clinical findings associated with diagnosing lumbar foraminal stenosis: systematic review
Source: BMC Musculoskelet Disord. 2026 Nov 3;27:969. doi: 10.1186/s12891-026-10614-5 (PMC13749674; doi:10.1186/s12891-026-10614-5)
Supplement: Supplementary file 3 [file 12891_2026_10614_MOESM3_ESM.docx]

# Appendix 3_ List of excluded studies/ reason for exclusion

1. Altan L, Metin Ökmen B, Tuncer T, Sindel D, Çay HF, Hepgüler S, Sarıkaya S, Ayhan F, Bal A, Bilgilisoy M, Çapkın E, Cerrahoğlu L, Çevik R, Dülgeroğlu D, Durmaz B, Duruöz T, Gürer G, Gürsoy S, Hizmetli S, Kaçar C, Kaptanoğlu E, Ecesoy H, Melikoğlu M, Nas K, Nur H, Özçakır Ş, Şahin N, Şahin Ö, Sarıdoğan M, Şendur ÖF, Sezer İ, Bozbaş GT, Tıkız C, Uğurlu H (2023) Correlation of clinical signs and magnetic resonance imaging findings in patients with lumbar spondylosis. Archives of Rheumatology 38:512-520. doi: 10.46497/ArchRheumatol.2023.9806

**Exclusion reason: Wrong patient population**

2. Annertz M, Holtås S, Cronqvist S, Jönsson B, Strömqvist B (1990) Isthmic lumbar spondylolisthesis with sciatica. MR imaging vs myelography. Acta Radiol 31:449-453 **Exclusion reason: No clinical data**
3. Aota Y, Niwa T, Yoshikawa K, Fujiwara A, Asada T, Saito T (2007) Magnetic resonance imaging and magnetic resonance myelography in the presurgical diagnosis of lumbar foraminal stenosis. Spine (Phila Pa 1976) 32:896-903. doi: 10.1097/01.brs.0000259809.75760.d5

**Exclusion reason: Wrong outcomes**

4. Ashida Y, Miki T, Kondo Y, Takebayashi T (2024) Influence of radiological factors, psychosocial factors, and central sensitization-related symptoms on clinical symptoms in patients with lumbar spinal canal stenosis. Journal of Back & Musculoskeletal Rehabilitation 37:369-377. doi: 10.3233/BMR-230093

**Exclusion reason: Wrong patient population**

5. Barrak AAZ (2019) Clinical and MRI evaluation of degenerative lumbar spine stenosis. Annals of Tropical Medicine and Public Health 22

**Exclusion reason: No fulltext available**

6. Beattie PF, Meyers SP, Stratford P, Millard RW, Hollenberg GM, Beattie PF, Meyers SP, Stratford P, Millard RW, Hollenberg GM (2000) Associations between patient report of symptoms and anatomic impairment visible on lumbar magnetic resonance imaging. Spine (03622436) 25:819-828. doi: 10.1097/00007632-200004010-00010

**Exclusion reason: Wrong patient population**

7. Bertilson BC, Brosjö E, Billing H, Strender LE, Bertilson BC, Brosjö E, Billing H, Strender L-E (2010) Assessment of nerve involvement in the lumbar spine: agreement between magnetic resonance imaging, physical examination and pain drawing findings. BMC Musculoskeletal Disorders 11:202-202. doi: 10.1186/1471-2474-11-202

**Exclusion reason: Wrong patient population**

8. Bhujade R, Shrivastva A, Kalavatia I, Baghel AS (2022) Correlation of conduction studies of tibial and superficial peroneal nerves with structural abnormalities diagnosed on magnetic resonance imaging in lumbar canal stenosis. European Journal of Molecular and Clinical Medicine 9:643-647

**Exclusion reason: No clinical data**

9. Cook C, Brown C, Michael K, Isaacs R, Howes C, Richardson W, Roman M, Hegedus E (2011) The clinical value of a cluster of patient history and observational findings as a diagnostic support tool for lumbar spine stenosis. Physiother Res Int 16:170-178. doi: 10.1002/pri.500

**Exclusion reason: Wrong patient population**

10. de Graaf I, Prak A, Bierma-Zeinstra S, Thomas S, Peul W, Koes B (2006) Diagnosis of lumbar spinal stenosis: a systematic review of the accuracy of diagnostic tests. Spine (Phila Pa 1976) 31:1168-1176. doi: 10.1097/01.brs.0000216463.32136.7b

**Exclusion reason: Wrong study design**

11. Di Muria GV, Stringa G, Marcucci M, Pitto RP (1992) Diagnosis, surgical indications and treatment of lumbar stenosis. Chir Organi Mov 77:51-54

**Exclusion reason: No fulltext available**

12. Dutta S, Bhave A, Patil S (2016) Correlation of 1.5 Tesla Magnetic Resonance Imaging with Clinical and Intraoperative Findings for Lumbar Disc Herniation. Asian Spine J 10:1115-1121. doi: 10.4184/asj.2016.10.6.1115

**Exclusion reason: Wrong patient population**

13. Farshad M, Sutter R, Hoch A (2018) Severity of foraminal lumbar stenosis and the relation to clinical symptoms and response to periradicular infiltration-introduction of the "melting sign". Spine J 18:294-299. doi: 10.1016/j.spinee.2017.07.176

**Exclusion reason: Wrong patient population**

14. Fushimi Y, Otani K, Tominaga R, Nakamura M, Sekiguchi M, Konno SI (2021) The association between clinical symptoms of lumbar spinal stenosis and MRI axial imaging findings. Fukushima J Med Sci 67:150-160. doi: 10.5387/fms.2021-22

**Exclusion reason: No clinical data**

15. Goh KJ, Khalifa W, Anslow P, Cadoux-Hudson T, Donaghy M (2004) The clinical syndrome associated with lumbar spinal stenosis. Eur Neurol 52:242-249. doi: 10.1159/000082369

**Exclusion reason: Wrong patient population**

16. Haig AJ, Geisser ME, Tong HC, Yamakawa KS, Quint DJ, Hoff JT, Chiodo A, Miner JA, Phalke VV (2007) Electromyographic and magnetic resonance imaging to predict lumbar stenosis, low-back pain, and no back symptoms. J Bone Joint Surg Am 89:358-366. doi: 10.2106/jbjs.E.00704

**Exclusion reason: Wrong patient population**

17. Haig AJ, Tong HC, Yamakawa KS, Quint DJ, Hoff JT, Chiodo A, Miner JA, Choksi VR, Geisser ME, Parres CM (2006) Spinal stenosis, back pain, or no symptoms at all? A masked study comparing radiologic and electrodiagnostic diagnoses to the clinical impression. Arch Phys Med Rehabil 87:897-903. doi: 10.1016/j.apmr.2006.03.016

**Exclusion reason: Wrong patient population**

18. Hellinger S, Lewandrowski KU (2020) Clinical outcomes with endoscopic resection of lumbar extradural cysts. J Spine Surg 6:S133-s144. doi: 10.21037/jss.2019.08.08

**Exclusion reason: Wrong outcomes**

19. Hughes SW, Hellyer PJ, Sharp DJ, Newbould RD, Patel MC, Strutton PH (2019) Diffusion tensor imaging reveals changes in microstructural integrity along compressed nerve roots that correlate with chronic pain symptoms and motor deficiencies in elderly stenosis patients. Neuroimage Clin 23:101880. doi: 10.1016/j.nicl.2019.101880

**Exclusion reason: No clinical data**

20. Hussein HM, Kamel E, Ragab M, Elerian A (2024) Diagnostic Accuracy of the Passive Straight Leg Raise Test in Detecting Compression of the Lower Lumbar Nerve Roots. Internet Journal of Allied Health Sciences & Practice 22:1-7

**Exclusion reason: Wrong patient population**

21. Hutchins J, Hebelka H, Lagerstrand K, Brisby H (2022) A systematic review of validated classification systems for cervical and lumbar spinal foraminal stenosis based on magnetic resonance imaging. Eur Spine J 31:1358-1369. doi: 10.1007/s00586-022-07147-5

**Exclusion reason: Wrong study design**

22. Iversen T, Solberg TK, Romner B, Wilsgaard T, Nygaard O, Waterloo K, Brox JI, Ingebrigtsen T, Nygaard Ø (2013) Accuracy of physical examination for chronic lumbar radiculopathy. BMC Musculoskeletal Disorders 14:206-206. doi: 10.1186/1471-2474-14-206

**Exclusion reason: Wrong patient population**

23. Jo N-G, Ko M-H, Won YH, Park S-H, Kim G-W, Seo J-H (2021) Diagnostic Implication and Clinical Relevance of Dermatomal Somatosensory Evoked Potentials in Patients with Radiculopathy: A Retrospective Study. Pain Research & Management:1-7. doi: 10.1155/2021/8850281

**Exclusion reason: No clinical data**

24. Jönsson B, Strömqvist B (1995) Motor affliction of the L5 nerve root in lumbar nerve root compression syndromes. Spine (03622436) 20:2012-2015

**Exclusion reason: No clinical data**

25. Jujjavarapu C, Pejaver V, Cohen TA, Mooney SD, Heagerty PJ, Jarvik JG (2022) A Comparison of Natural Language Processing Methods for the Classification of Lumbar Spine Imaging Findings Related to Lower Back Pain. Academic Radiology 29:S188-S200. doi: 10.1016/j.acra.2021.09.005

**Exclusion reason: No clinical data**

26. Kang DH, Baek J, Chang BS, Kim H, Hong SH, Chang SY (2024) Effects of the Severity of Stenosis on Clinical Outcomes of Indirect Decompression Using Oblique Lumbar Interbody Fusion. J Clin Med 13. doi: 10.3390/jcm13154421

**Exclusion reason: Wrong outcomes**

27. Kapellen PJ, Beall DP (2010) Imaging evaluation of low back pain: important imaging features associated with clinical symptoms. Seminars in Roentgenology 45:218-225. doi: 10.1053/j.ro.2009.12.001

**Exclusion reason: No clinical data**

28. Karppinen J, Malmivaara A, Tervonen O, Pääkkö E, Kurunlahti M, Syrjälä P, Vasari P, Vanharanta H (2001) Severity of symptoms and signs in relation to magnetic resonance imaging findings among sciatic patients. Spine (Phila Pa 1976) 26:E149-154. doi: 10.1097/00007632-200104010-00015

**Exclusion reason: Wrong patient population**

29. Katz JN, Zimmerman ZE, Mass H, Makhni MC (2022) Diagnosis and Management of Lumbar Spinal Stenosis: A Review. Jama 327:1688-1699. doi: 10.1001/jama.2022.5921

**Exclusion reason: Wrong study design**

30. Konno S, Hayashino Y, Fukuhara S, Kikuchi S, Kaneda K, Seichi A, Chiba K, Satomi K, Nagata K, Kawai S (2007) Development of a clinical diagnosis support tool to identify patients with lumbar spinal stenosis. Eur Spine J 16:1951-1957. doi: 10.1007/s00586-007-0402-2

**Exclusion reason: Wrong patient population**

31. Kuittinen P, Sipola P, Aalto TJ, Määttä S, Parviainen A, Saari T, Sinikallio S, Savolainen S, Turunen V, Kröger H, Airaksinen O, Leinonen V (2014) Correlation of lateral stenosis in MRI with symptoms, walking capacity and EMG findings in patients with surgically confirmed lateral lumbar spinal canal stenosis. BMC Musculoskelet Disord 15:247. doi: 10.1186/1471-2474-15-247

**Exclusion reason: Wrong outcomes**

32. Lau Y, Lee R, Griffith J, Chan C, Law S, Kwok K, Lau YYO, Lee RKL, Griffith JF, Chan CLY, Law SW, Kwok KO (2017) Changes in dural sac caliber with standing MRI improve correlation with symptoms of lumbar spinal stenosis. European Spine Journal 26:2666-2675. doi: 10.1007/s00586-017-5211-7

**Exclusion reason: Wrong patient population**

33. Lewandrowski KU, Lewandrowski KU, De Carvalho PST, De Carvalho P, Yeung A, Yeung A (2020) Minimal clinically important difference in patient-reported outcome measures with the transforaminal endoscopic decompression for lateral recess and foraminal stenosis. International Journal of Spine Surgery 14:254-266. doi: 10.14444/7034

**Exclusion reason: No clinical data**

34. Lin JH, Hsieh YC, Chen YC, Wang Y, Chen CC, Chiang YH (2017) Diagnostic accuracy of standardised qualitative sensory test in the detection of lumbar lateral stenosis involving the L5 nerve root. Sci Rep 7:10598. doi: 10.1038/s41598-017-10641-2

**Exclusion reason: Wrong outcomes**

35. Lin YT, Wang JS, Hsu WE, Lin YH, Wu YC, Chen KH, Pan CC, Lee CH (2023) Correlation of Foraminal Parameters with Patient-Reported Outcomes in Patient with Degenerative Lumbar Foraminal Stenosis. J Clin Med 12. doi: 10.3390/jcm1202047

**Exclusion reason: No clinical data**

36. Mataki K, Koda M, Shibao Y, Kumagai H, Nagashima K, Miura K, Noguchi H, Funayama T, Abe T, Yamazaki M (2020) New methods for diagnosing lumbar foraminal stenosis using dynamic digital tomosynthesis radiculography. J Clin Neurosci 77:106-109. doi: 10.1016/j.jocn.2020.05.011

**Exclusion reason: Wrong outcomes**

37. Naoto T, Osamu S, Kazuki K, Ryosuke M, Shin-ichi K (2016) Classifying patients with lumbar spinal stenosis using painDETECT: a cross-sectional study. BMC Family Practice 17:1-8. doi: 10.1186/s12875-016-0486-z

**Exclusion reason: Wrong patient population**

38. Orita S, Inage K, Eguchi Y, Kubota G, Aoki Y, Nakamura J, Matsuura Y, Furuya T, Koda M, Ohtori S (2016) Lumbar foraminal stenosis, the hidden stenosis including at L5/S1. Eur J Orthop Surg Traumatol 26:685-693. doi: 10.1007/s00590-016-1806-7

**Exclusion reason: No clinical data**

39. Park HJ, Kim SS, Lee SY, Park NH, Rho MH, Hong HP, Kwag HJ, Kook SH, Choi SH (2012) Clinical correlation of a new MR imaging method for assessing lumbar foraminal stenosis. AJNR Am J Neuroradiol 33:818-822. doi: 10.3174/ajnr.A2870

**Exclusion reason: No clinical data**

40. Peene L, Cohen SP, Kallewaard JW, Wolff A, Huygen F, Gaag Avd, Monique S, Vissers K, Gilligan C, Van Zundert J, Van Boxem K (2024) 1. Lumbosacral radicular pain. Pain Practice 24:525-552. doi: 10.1111/papr.13317

**Exclusion reason: Wrong study design**

41. Rabin A, Gerszten PC, Karausky P, Bunker CH, Potter DM, Welch WC (2007) The sensitivity of the seated straight-leg raise test compared with the supine straight-leg raise test in patients presenting with magnetic resonance imaging evidence of lumbar nerve root compression. Arch Phys Med Rehabil 88:840-843. doi: 10.1016/j.apmr.2007.04.016

**Exclusion reason: Wrong patient population**

42. Rainville J, Jouve C, Finno M, Limke J (2003) Comparison of four tests of quadriceps strength in L3 or L4 radiculopathies. Spine (03622436) 28:2466-2471. doi: 10.1097/01.brs.0000090832.38227.98
43. Rainville J, Lopez E (2013) Comparison of radicular symptoms caused by lumbar disc herniation and lumbar spinal stenosis in the elderly. Spine (03622436) 38:1282-1287. doi: 10.1097/BRS.0b013e31828f463e

**Exclusion reason: Wrong patient population**

44. Rajjoub R, Arroyave JS, Zaidat B, Ahmed W, Mejia MR, Tang J, Kim JS, Cho SK (2024) ChatGPT and its Role in the Decision-Making for the Diagnosis and Treatment of Lumbar Spinal Stenosis: A Comparative Analysis and Narrative Review. Global Spine Journal 14:998-1017. doi: 10.1177/21925682231195783

**Exclusion reason: Wrong patient population**

45. Sasaki K (1995) Magnetic resonance imaging findings of the lumbar root pathway in patients over 50 years old. Eur Spine J 4:71-76. doi: 10.1007/bf00278915

**Exclusion reason: Wrong outcomes**

46. Schroeder GD, Kurd MF, Vaccaro AR (2016) Lumbar Spinal Stenosis: How Is It Classified? J Am Acad Orthop Surg 24:843-852. doi: 10.5435/jaaos-d-15-00034

**Exclusion reason: No clinical data**

47. Shi Y, Zou Y, Feng Y, Dou W, Ding H, Wang C, Zhao F, Shi H (2020) A quantitative and clinical evaluation of nerve roots in lumbosacral radiculopathy using diffusion tensor imaging. Japanese Journal of Radiology 38:222-230. doi: 10.1007/s11604-019-00913-4

**Exclusion reason: Wrong patient population**

48. Sipola P, Leinonen V, Niemeläinen R, Aalto T, Vanninen R, Manninen H, Airaksinen O, Battié MC (2011) Visual and quantitative assessment of lateral lumbar spinal canal stenosis with magnetic resonance imaging. Acta Radiol 52:1024-1031. doi: 10.1258/ar.2011.110083

**Exclusion reason: No fulltext available**

49. Splettstößer A, Khan MF, Zimmermann B, Vogl TJ, Ackermann H, Middendorp M, Maataoui A (2017) Correlation of lumbar lateral recess stenosis in magnetic resonance imaging and clinical symptoms. World J Radiol 9:223-229. doi: 10.4329/wjr.v9.i5.223

**Exclusion reason: Wrong outcomes**

50. Suri P, Rainville J, Katz JN, Jouve C, Hartigan C, Limke J, Pena E, Li L, Swaim B, Hunter DJ (2011) The accuracy of the physical examination for the diagnosis of midlumbar and low lumbar nerve root impingement. Spine (Phila Pa 1976) 36:63-73. doi: 10.1097/BRS.0b013e3181c953cc

**Exclusion reason: Wrong patient population**

51. Takahashi K, Yadav AK, Hashimoto K, Tsubakino T, Aizawa T, Tanaka Y (2022) Foraminal Stenosis at L5-S1 as an Overlooked Pathology of Bilateral Radiculopathy: A Case Series. J Orthop Case Rep 12:13-18. doi: 10.13107/jocr.2022.v12.i06.2846

**Exclusion reason: Wrong study design**

52. Tomkins-Lane CC, Holz SC, Yamakawa KS, Phalke VV, Quint DJ, Miner J, Haig AJ (2012) Predictors of walking performance and walking capacity in people with lumbar spinal stenosis, low back pain, and asymptomatic controls. Arch Phys Med Rehabil 93:647-653. doi: 10.1016/j.apmr.2011.09.023

**Exclusion reason: Wrong patient population**

53. Trainor K, Pinnington MA (2011) Reliability and diagnostic validity of the slump knee bend neurodynamic test for upper/mid lumbar nerve root compression: a pilot study. Physiotherapy 97:59-64. doi: 10.1016/j.physio.2010.05.004

**Exclusion reason: Wrong patient population**

54. Verwoerd A, Mens J, el Barzouhi A, Peul W, Koes B, Verhagen A, Verwoerd AJH, Peul WC, Koes BW, Verhagen AP (2016) A diagnostic study in patients with sciatica establishing the importance of localization of worsening of pain during coughing, sneezing and straining to assess nerve root compression on MRI. European Spine Journal 25:1389-1392. doi: 10.1007/s00586-016-4393-8

**Exclusion reason: Wrong patient population**

55. Visser LH, Nijssen PGN, Tijssen CC, van Middendorp JJ, Schieving J (2013) Sciatica-like symptoms and the sacroiliac joint: clinical features and differential diagnosis. European Spine Journal 22:1657-1664. doi: 10.1007/s00586-013-2660-5

**Exclusion reason: Wrong patient population**

56. Weber C, Giannadakis C, Rao V, Jakola AS, Nerland U, Nygaard Ø P, Solberg TK, Gulati S, Solheim O (2016) Is There an Association Between Radiological Severity of Lumbar Spinal Stenosis and Disability, Pain, or Surgical Outcome?: A Multicenter Observational Study. Spine (Phila Pa 1976) 41:E78-83. doi: 10.1097/brs.0000000000001166

**Exclusion reason: Wrong patient population**

57. Wells MD, Meyer AP, Emley M, Kong X, Sanchez R, Gozani SN, Wells MD, Meyer AP, Emley M, Kong X, Sanchez R, Gozani SN (2002) Detection of lumbosacral nerve root compression with a novel composite nerve conduction measurement...including commentary by Haig AJ. Spine (03622436) 27:2811-2819

**Exclusion reason: No fulltext available**

58. Xin Z, Huang P, Zheng G, Liao W, Zhang X, Wang Y (2020) Using a percutaneous spinal endoscopy unilateral posterior interlaminar approach to perform bilateral decompression for patients with lumbar lateral recess stenosis. Asian J Surg 43:593-602. doi: 10.1016/j.asjsur.2019.08.010

**Exclusion reason: No clinical data**

59. Yamada K, Aota Y, Higashi T, Ishida K, Niimura T, Konno T, Saito T (2015) Roentgenographic and computed tomographic findings in symptomatic lumbar foraminal stenosis. Eur Spine J 24:333-338. doi: 10.1007/s00586-014-3683-2

**Exclusion reason: Wrong outcomes**

60. Yoshiiwa T, Miyazaki M, Notani N, Ishihara T, Kawano M, Tsumura H (2016) Analysis of the Relationship between Ligamentum Flavum Thickening and Lumbar Segmental Instability, Disc Degeneration, and Facet Joint Osteoarthritis in Lumbar Spinal Stenosis. Asian Spine J 10:1132-1140. doi: 10.4184/asj.2016.10.6.1132

**Exclusion reason: No clinical data**

61. Yoshimoto M, Kawaguchi S, Takebayashi T, Isogai S, Kurata Y, Nonaka S, Oki G, Kosukegawa I, Yamashita T (2009) Diagnostic features of sciatica without lumbar nerve root compression. J Spinal Disord Tech 22:328-333. doi: 10.1097/BSD.0b013e31817dc46d

**Exclusion reason: Wrong patient population**

62. Abdulmahmod O F, Al-Antari M A, Kwon H, Habib A, Raza M, Kaplan M, Ertuğrul B, Akçin İ, Bütün E, Gu Y. H. (2026) Medical Spine Sagittal MRI Dataset for Segmentation and Foraminal Stenosis detection. Sci Data 13:1. doi:10.1038/s41597-026-07138-x

**Exclusion reason: No clinical data**

63. Barraza-Silva JA, Sauri-Barraza JC. (2024) Index for static and dynamic measurements of the lumbar foramina in patients with foraminal stenosis. Acta Ortopédica Mexicana 38: 285-290. doi: 10.35366/117376

**Exclusion reason: No clinical data**

64. Bingi S,Vijaypavan Kumar D, Idris Hussain S. (2025) A Retrospective Analysis of MRI Findings in Patients Presenting with Chronic Low Back Pain: Patterns and Clinical Correlation. Eur J Cardiovasc Med 15:900-4. doi:10.5083/ejcm/25-05-172

**Exclusion reason: No clinical data**

65. Dhuria1 A, Kumar Singh S, Pachauria P. (2025). Impact of Stenosis and Neural Foraminal Compromise on Low Back Pain and Postural Alignment. Int J Health Sci Res 5:55-9. doi: 10.52403/ijhsr.20250507

**Exclusion reason: Wrong patient population**

66. Eldanasory O A. (2026) Omar sign update for the clinical diagnosis of unilateral lumbar foraminal stenosis. Egypt J Neurosurg 41:61-5. doi:10.1186/s41984-026-00574-2

**Exclusion reason: Wrong patient population**

67. Menon V, Vishnu Mane A, Borde M, Rajale S, Kanade U, Datey T, Kumar A. (2026) Does magnetic resonance imaging morphology correlate with preoperative neurological status in operatively treated lumbar canal stenosis? A retrospective study. Asian Spine Journal 3:1-7. doi:10.31616/asj.2025.0451

**Exclusion reason: Wrong patient population**

68. Sá Silva J, Pereira A, Abreu V, Filipe J P (2025) Inter-observer variability in the classification of lumbar foraminal stenosis in magnetic resonance imaging using different evaluation scales. Eur Spine J 34:869-873. doi: 10.1007/s00586-024-08612-z

**Exclusion reason: No clinical data**

69. Sugiura K, Payne C, Nguyen T, Tran, Leyendecker J, Ogunlade‖, LaVanne M, Derman P D, Quon R, Albert E, Hofstetter C P. (2025) Transforaminal Full-Endoscopic Surgery for Lumbar Foraminal Pathologies: A Comparative Clinical Effectiveness Study. Neurosurgery 96: S51-S62. doi: 10.1227/neu.0000000000003337

**Exclusion reason: No clinical data**
